# Dietary Antioxidant Intake and Human Papillomavirus Infection: Evidence from a Cross-Sectional Study in Italy

**DOI:** 10.3390/nu12051384

**Published:** 2020-05-12

**Authors:** Martina Barchitta, Andrea Maugeri, Claudia La Mastra, Maria Clara La Rosa, Giuliana Favara, Roberta Magnano San Lio, Antonella Agodi

**Affiliations:** Department of Medical and Surgical Sciences and Advanced Technologies “GF Ingrassia”, University of Catania, 95123 Catania, Italy; martina.barchitta@unict.it (M.B.); andrea.maugeri@unict.it (A.M.); claudia.lamastra@unict.it (C.L.M.); mariclalarosa@gmail.com (M.C.L.R.); giuliana.favara@gmail.com (G.F.); robimagnano@gmail.com (R.M.S.L.)

**Keywords:** nutritional epidemiology, antioxidants, human papillomavirus, cervical cancer, zinc

## Abstract

Several lines of evidence suggested that antioxidants might play a protective role against high-risk human papillomavirus (hrHPV) infection and cervical cancer. However, the effect of combined intake of antioxidants has not been investigated thus far. The current cross-sectional study aimed to understand the relationship between dietary antioxidant intake and the risk of high-risk HPV (hrHPV) infection among 251 Italian women with normal cervical cytology. Women were tested for hrHPV using the Digene HC2 HPV DNA Test. Dietary antioxidant intakes were assessed using a semi-quantitative food frequency questionnaire, and a Composite Dietary Antioxidant Index (CDAI) was constructed on the basis of zinc, selenium, manganese, vitamin A, vitamin C, vitamin E, carotenoid, and flavonoid intake. Logistic regression analysis was used to assess odds ratio (OR) and 95% confidence interval (95% CI) for the associations of antioxidant intakes or CDAI with hrHPV status, adjusting for age, smoking status, body mass index, parity, educational level, marital status, and use of multivitamins and oral contraceptives. We first observed that hrHPV-positive women (*n* = 84) reported lower intake of zinc, manganese, and vitamins A and C than non-infected women. Specifically, we found a negative association between dietary intake of zinc and hrHPV-positive status when all antioxidants were considered simultaneously (OR = 0.46; 95% CI = 0.27–0.80; *p* = 0.006). With respect to cumulative dietary antioxidant intake, we demonstrated that women with high CDAI (third tertile) had lower odds of being hrHPV-positive than those with low CDAI (first tertile) (OR = 0.39; 95% CI = 0.18–0.85; *p* = 0.018). To our knowledge, this is the first study demonstrating that a diet based on the combined intake of nutrients with antioxidant properties might reduce the risk of hrHPV infection. However, further research is needed to understand whether dietary antioxidant intake is associated with hrHPV infection or its persistence.

## 1. Introduction

Papillomaviruses are ubiquitous DNA viruses that are transmitted primarily by direct physical contact, thus being able to cause benign flat, protruding warts or, occasionally, cancer. Among more than 200 human papillomavirus (HPV) genotypes, the high-risk HPV (hrHPV) types are sexually transmitted and are typically controlled immunologically within 1–2 years [1]. Yet, if infection persists, it can cause cervical cancer, one of the most common cancers in women worldwide [2]. In fact, it is by far the most common HPV-related cancer [3], with approximately 570,000 cases and 310,000 deaths in 2018 [2]. Nowadays, the development of effective preventive strategies based on vaccines and screening are radically changing the landscape of HPV-related cancers [4]. However, globally, the effective implementation of HPV vaccination and screening still remains a challenge for public health.

HPV infection is necessary but may not be sufficient for the development of cervical cancer, and hence other cofactors may modulate the progression of HPV infection to cervical cancer. For instance, mounting evidence has shown that the consumption of “healthy” foods (e.g., fruits and vegetables) and the intake of certain nutrients (e.g., antioxidants, folates, and minerals) are associated with a decreased risk of HPV infection, precursor lesions, and cervical cancer [5,6,7,8,9,10,11,12,13,14].

Specifically, it has been demonstrated that cellular oxidative environment and reactive oxygen species (ROS) production play a crucial role in cell signaling and homeostasis in response to pathogens. Given that viruses replicate in living cells, oxidative stress might induce an antiviral state that limits the viral replication [15]. Although oxidative stress represents a mechanism underpinning the host cellular response to viral infection, it has also been observed that some viruses could thrive in an oxidative environment [15]. With respect to HPV, it has been demonstrated that the virus induces oxidative stress and DNA damage, leading to cancer progression [16,17]. Notably, several lines of evidence have proposed an inverse relationship between antioxidant plasma concentrations and the risk of HPV infection [18,19,20,21,22,23,24]. However, findings in support of a relationship between dietary intake of antioxidants and HPV status are currently inconclusive [25]. For instance, non-significant or weak protective effects of carotenoids against HPV persistence have been reported by observational studies [25]. In line with this, several randomized controlled trials (RCTs) did not demonstrate benefit from oral administration of beta-carotene against cervical cancer progression [26,27]. With regards to retinoids, evidence for a protective effect of dietary intake against HPV persistence is inconclusive but probable for cervical cancer progression [25]. Similarly, observational evidence for benefits from vitamin C and E against HPV persistence and cervical cancer is plausible [25], although no trials have demonstrated a protective effect of oral supplementation [28,29]. Despite the above findings, however, it is important to mention that some preclinical in vivo studies suggest how antioxidants could promote tumor growth and metastasis [30,31,32]. Some RCTs have also reported that antioxidant supplementation during cancer treatment might alter the effectiveness of therapies, with the worst outcome being among smokers [33].

In a context where findings are still inconclusive, to our knowledge, no studies have investigated the association of cumulative dietary antioxidant intake with HPV infection and/or with cervical cancer risk. Yet, the evaluation of the cumulative effect of antioxidants—for instance, using the Composite Dietary Antioxidant Index (CDAI)—seems a promising approach to estimate the effect of antioxidant intake on several diseases [34]. Accordingly, we have hypothesized an association between CDAI and HPV infection in women without cervical cancer or precursor lesions. Thus, in the present cross-sectional study, we assessed dietary intake of antioxidants and CDAI among women with normal cervical cytology from Catania, Italy. Next, we evaluated differences in dietary antioxidant intake and CDAI between hrHPV-positive and hrHPV-negative women to test the association between cumulative antioxidant intake and hrHPV infection.

## 2. Materials and Methods

### 2.1. Study Design

In the present cross-sectional analysis, we used data from a previously published study on women who referred to the Cervical Cancer Screening Unit of the Azienda Sanitaria Provinciale (ASP) in Catania, Italy [5,35]. The study design, protocols, and population characteristics have been fully described elsewhere [5,35]. The study was approved by the ethics committee of the involved Institution (CE Catania 2; Protocol Number 227/BE and 275/BE) and conducted in line with the Declaration of Helsinki. Women were informed about study design, procedures, and objectives of the study and gave their consent to participate. In brief, during a three-year period, the study recruited 539 women with an abnormal Papanicolaou (PAP) test prior to undergoing treatment. In the current analysis, we included all women with normal cervical cytology and complete assessment of dietary antioxidant intake, as well as sociodemographic and behavioral information. Each woman was tested for hrHPV using the Digene HC2 HPV DNA Test (Qiagen, Milan, Italy), and categorized as hrHPV-positive or hrHPV-negative.

### 2.2. Assessment of Socio-Demographic and Behavioral Information

Data on sociodemographic characteristics and behavioral factors were collected using a structured questionnaire. Marital status was classified as living in a couple (including marriage and other relationships) or living alone (including being single, divorced, or widowed). Women were also classified as employed (full-time or part-time employment) or unemployed (including housewives). Education was categorized into low (primary education) or medium–high (secondary and tertiary education) educational level. Women were classified according to their smoking status as current, former, or never smokers. Body mass index (BMI) was self-reported and defined according to the World Health Organization [36]. Women also reported their use of oral contraceptives and supplements (both multivitamin and/or multimineral supplements) at the time of recruitment. However, no information about the regimen and dose of specific antioxidant administration were available.

### 2.3. Assessment of Dietary Antioxidant Intake

Dietary assessment and analysis were performed as previously described [5,37,38,39,40]. In brief, dietary intakes of eight antioxidants (zinc, selenium, manganese, vitamin A, vitamin C, vitamin E, carotenoids, and flavonoids) and total energy intake were collected by a semi-quantitative Food Frequency Questionnaire (FFQ), referring to 1 month prior to the recruitment. Women in the 5th and 95th percentiles for total energy intake were considered as outliers and excluded from the current analysis. To account for differences in total energy intake, dietary antioxidant intake was adjusted by calorie intake using the residual method [41]. We also adapted the CDAI, proposed by Wright and colleagues [34], to investigate the association of cumulative dietary antioxidant intake with hrHPV infection. The CDAI was computed on the basis of the dietary intake of eight antioxidants (i.e., zinc, selenium, manganese, vitamin A, vitamin C, vitamin E, carotenoids, and flavonoids) derived from the FFQ. In particular, the CDAI was obtained by summing the z-score of dietary intakes of the eight antioxidants, and then classified according to the tertile distribution. Using the CDAI, rather than specific antioxidant intakes, allows for the investigation of the cumulative effect of antioxidant in several physiological and pathological conditions [34].

### 2.4. Statistical Analyses

Statistical analyses were conducted using the SPSS software (version 22.0, SPSS, Chicago, IL, USA). The study population was described using frequency and percentage for qualitative characteristics, and median with interquartile range (IQR) for quantitative data. According to the Kolmogorov–Smirnov test for normality, continuous variables underlying non-normal distribution were compared using the Mann–Whitney *U* test for comparisons between hrHPV-positive and -negative women or using the Kruskal–Wallis test for comparisons across CDAI categories. Categorical variables were compared using the chi-squared test. The Bonferroni method was used to correct for multiple comparisons. Linear regression models were used to evaluate the association of social and behavioral factors with CDAI. Logistic regression models were used to investigate the associations of dietary antioxidant intake with hrHPV status. The models included the standardized dietary intake of antioxidants (i.e., either separately or simultaneously) or the CDAI (i.e., both as continuous and as categorical) as independent variables, and were adjusted for age, smoking status, BMI, parity, educational level, marital status, and use of supplements and oral contraceptives. All statistical tests were two-sided, and *p*-value <0.05 was considered as statistically significant.

## 3. Results

### 3.1. Dietary Intake of Antioxidants According to HPV Status

The current study included 251 women (median age = 46.5 years, IQR = 19.0) with normal cervical cytology who satisfied the selection criteria. The characteristics of women according to their hrHPV diagnosis were fully reported elsewhere [5]. In brief, the 84 hrHPV-positive women (33.5%) were younger (*p* < 0.001), more likely to be smokers (*p* = 0.003), and less likely to be obese (*p* < 0.004) and to have children (*p* < 0.001) than their hrHPV-negative counterparts.

Table 1 displays total energy intake and dietary intake of antioxidants according to hrHPV diagnosis. In general, we found that hrHPV-positive women reported a lower total energy intake compared with their hrHPV-negative counterpart. This was consistent with a lower intake of zinc, manganese, and vitamins A and C among hrHPV-positive women. In particular, we found a negative association between dietary intake of zinc and hrHPV-positive status (odds ratio (OR) = 0.49; 95% CI = 0.27–0.89; *p* = 0.018, for one-unit increase in the standardized intake) after adjusting for age, smoking status, BMI, parity, educational level, marital status, and use of supplements and oral contraceptives. Notably, this negative relationship remained significant when considering the effect of all antioxidants simultaneously (OR = 0.46; 95% CI = 0.27–0.80; *p* = 0.006, for one-unit increase in the standardized intake).

### 3.2. Population Characteristics According to the Composite Dietary Antioxidant Index

To evaluate the synergistic effect of dietary antioxidants, we first computed the CDAI and ranked each woman according to the tertile distribution. In general, we observed that women with higher CDAI (third tertile) were older and more likely to live in a couple, to be less educated, and to have children, but less likely to be a normal weight and to use oral contraceptives than those with lower CDAI (first tertile) (Table 2). However, only age maintained a positive association with CDAI when all these factors were evaluated simultaneously (β = 0.34; Standard error, SE = 0.04; *p* < 0.001).

### 3.3. Association of Composite Dietary Antioxidant Index with HPV Status

**Figure 1:**
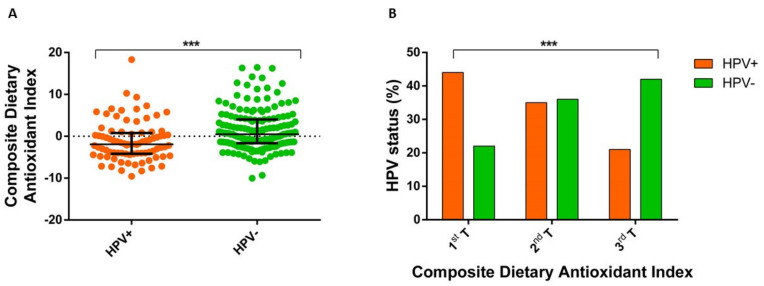
Association between the Composite Dietary Antioxidant Index and high-risk human papillomavirus (HPV) status. (**A**) Comparison of Composite Dietary Antioxidant Index between high-risk HPV-positive and -negative women using the Mann–Whitney *U* test (*** *p* < 0.001); (**B**) Distribution of women by high-risk HPV status and tertiles of Composite Dietary Antioxidant Index (*** *p* < 0.001 based on the chi-squared test).

Next, we found that CDAI was lower among hrHPV-positive women when compared with their negative counterpart (Figure 1A). In line with this evidence, we also reported that the proportion of hrHPV-positive women decreased with increasing CDAI (Figure 1B). Interestingly, we found that the odds of being hrHPV-positive decreased by 8% for each one-unit increase in CDAI (OR = 0.92; 95% CI = 0.86–0.98; *p* = 0.020), after adjusting for age, smoking status, BMI, parity, educational level, marital status, and use of supplements and oral contraceptives. Similarly, women with high CDAI (third tertile) had lower odds of being hrHPV-positive than those with low CDAI (first tertile) (OR = 0.39; 95% CI = 0.18–0.85; *p* = 0.018), after adjusting for covariates.

## 4. Discussion

Several lines of evidence have already shown how dietary habits might affect the risk of cancer in women [42,43]. With respect to cervical cancer, however, persistent hrHPV infection is an essential factor for the progression of precursor lesions to cancer [4]. For this reason, there is also the need for evaluating other factors, in particular the association between diet and hrHPV infection. In a previous study, we demonstrated the dual effect of diet on the risk of hrHPV infection—on one hand, the risk increased among women with high adherence to a Western dietary pattern, on the other hand, it decreased among those who adhered to the Mediterranean diet [5]. In the current study on the same population, we intended to deepen our analysis evaluating whether dietary antioxidant intake was associated with hrHPV status among women with normal cervical cytology. As summarized by Garcıa-Closas, in fact, antioxidants might exert a protective effect against cervical cancer progression by modulating immune response, viral replication, and gene expression [25]. In line with this, a recent review by Chih and colleagues suggested that high levels of plasma antioxidants might enhance the clearance of hrHPV [44]. By contrast, previous studies reported inconsistent results about the effect of dietary antioxidant intake, probably due to differences in study design, populations, and methods used for dietary assessment. For instance, a case-control study by Giuliano and colleagues found that dietary intakes of b-cryptoxanthin, lutein, zeaxanthin, and vitamin C were negatively associated with the risk of HPV persistence [45]. Similarly, a cohort study reported a borderline significant association between dietary lutein intake and HPV persistence [46]. By contrast, in our study, no differences in dietary intake of carotenoids were evident between hrHPV-positive and -negative women. However, we observed a lower dietary intake of zinc, manganese, and vitamin A and C among women with hrHPV infection. Among these, dietary zinc intake maintained a negative association with the risk of hrHPV-positive status when all antioxidants were considered simultaneously. This finding supports the notion that zinc is involved in the immunomodulatory pathways—specifically, zinc intake and its deficiency might be associated with hypoplasia of lymphoid tissues followed by a reduction in the number and activity of T-helper and natural killer cells, antibody production, cell mediated immunity, and phagocytosis [47,48,49,50,51]. In line with this, a pilot study demonstrated that a zinc-based compound via intra-vaginal infusion might help the clearance of hrHPV from the uterine cervix among women with no evidence of high grade squamous intraepithelial lesions [52].

To our knowledge, our study is also the first that has evaluated the influence of a combination of dietary antioxidants on hrHPV status using the CDAI. This index—developed by Wright and colleagues—allows for the ranking of dietary antioxidant intake in relation to population norms and the evaluation of the overall impact of antioxidants on health outcomes [34,53]. Notably, in our study, women with higher dietary antioxidant intake had lower odds of being infected by hrHPV than those who reported a lower intake. The current lack of evidence supporting our findings, however, raises the need for further research on large and prospective cohorts. In particular, it is necessary to understand to what extent dietary intake of antioxidants could prevent HPV infection and promote its clearance. Indeed, HPV survives in a cellular oxidative environment and even leads to neoplastic changes by increasing oxidative stress and DNA damage [16,17]. For this reason, dietary antioxidant intake could exert a protective effect against HPV infection and persistence [25]. However, further investigations on the biological mechanisms underpinning the antioxidant effect against HPV should be encouraged. In this scenario, our study adds to the current knowledge about the association between dietary antioxidant intake and the risk of HPV infection. Our findings, if confirmed by large-scale prospective research, might inform novel preventive strategies against HPV infection on the basis of dietary recommendations, especially in regions where vaccination is not implemented yet or vaccination coverage levels are not sufficient for prevention of infections. It is important to clarify that our study does not intend to give recommendations regarding the use of antioxidant supplements. In fact, there is the need for RCTs to evaluate benefits and drawbacks of antioxidant supplementation in women at risk for cervical cancer.

Our study had some limitations that should be considered when interpreting our findings. First, the cross-sectional design and the absence of information about timing of infection did not allow us to determine the causality of the relationship, nor did it allow us to discriminate between hrHPV infection and persistence. Second, dietary assessment did not preclude measurement error and inaccuracy. Third, no information about dosage regimen of antioxidant supplementation was available. To partially address this issue, we adjusted our analysis for the use of supplements, which could also include antioxidants. We found no significant differences in the use of supplements according to hrHPV status and CDAI tertiles. In line with this, our findings remained significant after adjusting for the use of supplements and other covariates. However, we cannot completely exclude the effect of unmeasured residual factors that potentially affect dietary antioxidant intake and/or hrHPV status.

In conclusion, our study showed that dietary intake of zinc was negatively associated with the risk of hrHPV infection, probably due to its immunomodulatory properties. Moreover, to our knowledge, we demonstrated for the first time that the cumulative intake of antioxidants might be associated with a decreased risk of hrHPV infection. However, further large-scale prospective studies are encouraged to understand whether antioxidants exert their effect on hrHPV infection, rather than on its persistence.

## Figures and Tables

**Table 1:** Dietary intake of antioxidants according to high-risk human papillomavirus (HPV) status.

Dietary Intake	HPV-Negative (*n* = 167)	HPV-Positive (*n* = 84)	*p*-Value
Total energy intake, kcal	2080 (703)	1747 (722)	**<0.001**
Zinc, mg	9.21 (2.93)	7.60 (3.80)	**<0.001**
Selenium, μg	319.14 (485.53)	311.63 (461.46)	0.272
Manganese, mg	314.64 (93.03)	266.33 (101.19)	**<0.001**
Vitamin A, IU	1097.59 (538.14)	827.32 (586.10)	**0.002**
Vitamin C, mg	116.71 (107.55)	82.21 (84.34)	**0.001**
Vitamin E, mg	37.97 (23.44)	34.08 (20.49)	0.158
Carotenoids, μg	9267.17 (7369.62)	7749.60 (6973.24)	0.052
Flavonoids, μg	1624.20 (6850.78)	819.81 (4964.17)	0.163

Data are reported as median (interquartile range) and compared using the Mann–Whitney test. *p*-values < 0.05 are indicated in bold font.

**Table 2:** Characteristics of the study population according to tertile distribution of Composite Dietary Antioxidant Index.

Characteristics	Composite Dietary Antioxidant Index			*p*-Value
	First Tertile (*n* = 73)	Second Tertile (*n* = 89)	Third Tertile (*n* = 89)	
**Age, years**	31.0 (8.0)	41.0 (7.0)	52.0 (9.0)	**<0.001**
**Current smokers (%)**	40.3%	38.2%	25.8%	0.102
**BMI, kg/m^2^**	20.8 (4.2)	23.4 (4.8)	23.7 (5.4)	**<0.001**
**BMI Categories (%)**				
**Underweight**	12.3%	6.8%	1.1%	**<0.001**
**Normal weight**	74.0%	61.4%	55.7%	
**Overweight**	6.8%	18.2%	31.8%	
**Obese**	6.8%	13.6%	11.4%	
**Living in a couple (%)**	39.7%	61.8%	74.2%	**<0.001**
**Employed (%)**	46.6%	47.2%	40.4%	0.613
**Low educational level (%)**	26.0%	39.3%	47.2%	**0.022**
**Having children (%)**	50.7%	78.7%	91.0%	**<0.001**
**Use of oral contraceptive (%)**	16.4%	9.0%	4.5%	**0.036**
**Use of multivitamin supplements (%)**	8.2%	12.4%	16.9%	0.258

Data are reported as median (interquartile range) and compared using the Kruskal–Wallis test. *p*-values < 0.05 are indicated in bold font. BMI, body mass index
